# Recombination, cryptic clades and neutral molecular divergence of the microcystin synthetase (*mcy*) genes of toxic cyanobacterium *Microcystis aeruginosa*

**DOI:** 10.1186/1471-2148-9-115

**Published:** 2009-05-22

**Authors:** Yuuhiko Tanabe, Tomoharu Sano, Fumie Kasai, Makoto M Watanabe

**Affiliations:** 1 Graduate School of Life & Environmental Sciences, University of Tsukuba, 1-1-1 Tennodai, Tsukuba, Ibaraki 305-8572, Japan; 2 Laboratory of Intellectual Fundamentals for Environmental Studies, National Institute for Environmental Studies, 16-2 Onogawa, Tsukuba, Ibaraki 305-8506, Japan; 3 Environmental Biology Division, National Institute for Environmental Studies, 16-2 Onogawa, Tsukuba, Ibaraki 305-8506, Japan

## Abstract

**Background:**

The water-bloom-forming cyanobacterium *Microcystis aeruginosa *is a known producer of various kinds of toxic and bioactive chemicals. Of these, hepatotoxic cyclic heptapeptides microcystins have been studied most intensively due to increasing concerns for human health risks and environmental damage. More than 70 variants of microcystins are known, and a single microcystin synthetase (*mcy*) gene cluster consisting of 10 genes (*mcyA *to *mcyJ*) has been identified to be responsible for the production of all known variants of microcystins. Our previous multilocus sequence typing (MLST) analysis of the seven housekeeping genes indicated that microcystin-producing strains of *M. aeruginosa *are classified into two phylogenetic groups.

**Results:**

To investigate whether the *mcy *genes are genetically structured similarly as in MLST analysis of the housekeeping genes and to identify the evolutionary forces responsible for the genetic divergence of these genes, we used 118 *mcy*-positive isolates to perform phylogenetic and population genetic analyses of *mcy *genes based on three *mcy *loci within the *mcy *gene cluster (*mcyD*, *mcyG*, and *mcyJ*), none of which is involved in the production of different microcystin variants. Both individual phylogenetic analysis and multilocus genealogical analysis of the *mcy *genes divided our isolates into two clades, consistent with the MLST phylogeny based on seven housekeeping loci. No shared characteristics within each clade are known, and microcystin analyses did not identify any compositional trend specific to each clade. Statistical analyses for recombination indicated that recombination among the *mcy *genes is much more frequent within clades than between, suggesting that recombination has been an important force maintaining the cryptic divergence of *mcy *genes. On the other hand, a series of statistical tests provided no strong evidence for selection to explain the deep divergence of the *mcy *genes. Furthermore, analysis of molecular variance (AMOVA) indicated a low level of geographic structuring in the genetic diversity of *mcy*.

**Conclusion:**

Our phylogenetic analyses suggest that the *mcy *genes of *M. aeruginosa *are subdivided into two cryptic clades, consistent with the phylogeny determined by MLST. Population genetic analyses suggest that these two clades have primarily been maintained as a result of homology-dependent recombination and neutral genetic drift.

## Background

**Figure 1:**
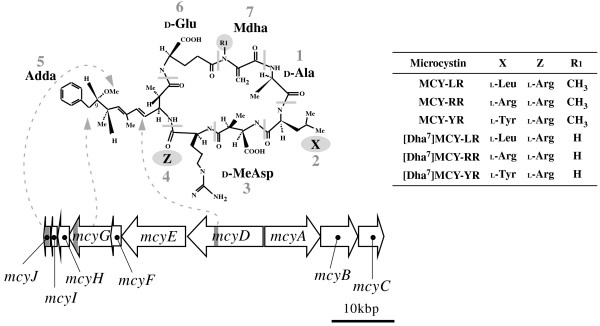
**Structure of the microcystin synthetase (*mcy*) gene and microcystins**. Structural representation of microcystin variants and the microcystin synthetase (*mcy*) gene cluster [5]. General numbering of amino acids is indicated in gray. Arrows indicate the proposed involvement of the product of each *mcy *marker locus in the incorporation and/or modification of each amino acid into the microcystin. Note that the amino acids (X and Z) and groups (R_1_) highlighted in gray are variable. Microcystin is abbreviated as "MCY" in the right-hand table. Abbreviations for three uncommon amino acids in microcystins are as follows: _D_-MeAsp, _D_-erythro-β-methylaspartic acid; Adda, (2*S*, 3*S*, 8*S*, 9*S*)-3-amino-9-methoxy-2,6,8-trimethyl-10-phenyldeca-4,6-dienoic acid; MdhA, *N*-methyldehydroalanine. The positions of PCR primers in the *mcy *genes are indicated by a gray box.

Microcystins are a family of cyclic heptapeptides consisting of seven characteristic amino acids (Fig. 1). Exposure to microcystins poses a severe health risk for both humans and animals, primarily because of hepatotoxicity. Accidental ingestion of water contaminated with microcystins causes acute hepatitis due to the inhibition of protein phosphatase 1 (PP1) and PP2A in hepatocytes, and the possible involvement of microcystins in tumor promotion has also been suggested [1]. Although a number of cyanobacterial genera (e.g., *Anabaena*, *Planktothrix, Nostoc*) are known to produce microcystins, the primary producer of microcystins is the water-bloom-forming cyanobacterium *Microcystis aeruginosa *that is often found in eutrophic freshwater environments such as ponds, lakes, and reservoirs worldwide.

More than 70 structural variants of microcystin with varying levels of toxicity have been reported [2]. Most of these variants differ from each other at the second (X) and/or fourth (Z) amino acid position in the cyclic heptapeptide. Another form of variant in which one or two amino acids are demethylated is also often encountered (Fig. 1). Many strains of *M. aeruginosa *are known to produce more than two variants of microcystins [3]. A single microcystin synthetase (*mcy*) gene cluster (Fig. 1) has been shown to be responsible for all structural variants of microcystins [4,5]. The product of the *mcy *gene cluster is a large multienzyme complex of mixed polyketide synthase (PKS) and non-ribosomal peptide synthetase (NRPS) modules.

The *mcy *gene cluster of *M. aeruginosa *comprises 10 genes, *mcyA *to *mcyJ*, nine of which encode catalytic domains for microcystin synthesis [4,5]. By contrast, the product of *mcyH *is hypothesized to be involved in intra- (or extra-) cellular transportation of microcystins [6]. As in other NRPSs, the products of the *mcy *gene cluster possess the same number of basic "modules" as the number of amino acids incorporated into the non-ribosomal peptide, synthesizing microcystins by a thiotemplate mechanism [7]. Each NRPS module contains an adenylation domain (A domain), a domain for specific amino acid activation, a condensation domain (C domain), a domain for specific amino acid recognition and peptide bond elongation, and a peptidyl carrier protein (PCP). Similarly, the PKS-coding components of the *mcy *gene cluster contain genes encoding type I PKS modules consisting of a β-ketoacyl synthase (KS domain), an acyltransferase (AT domain), β-ketoacyl reductase (KR domain), a dehydratase (DH domain), and an acyl carrier protein (ACP). Other optional domains for tailoring enzymes are also present in the *mcy *gene cluster.

Given that only a single *mcy *gene cluster is present in the genome of strains producing two or more variants of microcystins [4,5,8], it has been suggested that the structural variation of microcystins differing in amino acid composition is due to nonspecific amino acid recognition by A domains encoded by the *mcy *genes rather than differences in the primary structures of *mcy *[3]. Because the two NRPS modules encoded by the first part of *mcyB *(*mcyB1*) and *mcyC *are responsible for the variable amino acids in microcystins (X and Z, respectively), most previous work investigating the genetic diversity of *mcy *has focused on these two genes [3,9-11]. Interestingly, it has been demonstrated that gene conversion between A domains, but not C domains, encoded by *mcyB1 *and *mcyC*, can explain the difference in the production of specific microcystin variants in a number of strains [9,10]. Positive selection at the codon near the binding pocket of the A domain might have an impact on the genetic diversity of *mcyC *[11]. These results suggest that variation of microcystin composition might be genetically structured to some extent. An important finding has been the recombinational replacement of A domains encoded by *mcyB1 *with domains from phylogenetically distant origins, probably occurring as a result of horizontal transmission [10]. This finding questions the validity of using A domains, or other redundant domains of PKS and NRPS, to investigate the overall phylogenetic relationships of the *mcy *gene cluster. Moreover, it has also been demonstrated that both intra- and intergenic recombination significantly contributes to the overall genetic diversity of the *mcy *genes [12]. Recent occurrences of recombination can significantly bias the result of phylogenetic analysis because they decouple genetic similarity from evolutionary history. Therefore, multiple and conservative regions of the *mcy *locus should be used as genetic markers to understand better the evolutionary relationships and genetic diversity of the *mcy *genes as a whole.

Previously we demonstrated that toxic strains are divided into two lineages, group A and group B, based on multilocus phylogenetic analysis of the seven housekeeping genes (MLST) [13]. This finding raises the following two questions: 1) Are *mcy *genes structured similarly to the housekeeping genes? Or did they evolve independently from the other genes in the genome of *M. aeruginosa*? 2) Which evolutionary forces are responsible for the observed genetic divergence of the *mcy *genes? (e.g., are the *mcy *genes structured as a result of selective sweep or neutral genetic drift following geographic isolation?) To address these issues, we have analyzed the *mcy *genes from 118 toxic and non-toxic strains of *M. aeruginosa*.

We performed a suite of phylogenetic and population genetic analyses of the *mcy *genes using three "conservative" loci within the *mcy *gene cluster as markers (designated "*mcy*MLST" by analogy with MLST). MLST is a multilocus sequence-based genotyping protocol that has been widely used to investigate the population structures of a broad spectrum of microbial species ranging from bacteria and archaea to eukaryotic microbes [14,15]. A conventional MLST analysis based on the seven housekeeping loci [13] was also performed to compare it with the results of the *mcy*MLST analysis. We measured the microcystin composition of each strain to investigate the possible correlation between *mcy *genetic divergence and microcystin composition. Our results show how evolutionary forces, particularly recombination and genetic drift, have contributed to the genetic divergence of *mcy *genes.

## Methods

### Strains, culture, isolation and DNA extraction

The 196 strains of *M. aeruginosa *used in this study are listed in Additional file 1. They include 11 toxic strains that we previously characterized using *mcyA, mcyD, mcyG*, and *mcyJ *[12], 75 *mcyG*-positive and 78 *mcyG*-negative strains used in our previous report [13], and 32 toxic strains recently isolated in 2004–2006. The novel 32 strains were isolated by the following protocol. Colonies of *M. aeruginosa *were picked from field water samples by using a glass micropipette under a microscope, and cultured in a 1:1 mixture of liquid MA medium [16] and autoclaved field water filtrated using a nylon net filter with 0.22 μm pore size (Millipore). From these "crude" cultures, a single cell was picked to establish a clonal strain by the same micromanipulation procedure used in crude isolation except that MA medium was used for clonal cultures. Of the 196 strains, 164 are currently available at MCC-NIES (Tsukuba, Japan) and the 32 newly isolated strains will be available at MCC-NIES in the future. Cultures were grown in 10 ml of MA medium at 25°C for 1–3 weeks under a 12:12 L/D cycle with a photon density of 15 μmol m^-2^s^-1^. Genomic DNA was extracted and purified by a FastDNA^® ^kit (Q-BIOgene).

### Multilocus sequencing typing (MLST) of seven housekeeping genes

The published MLST protocol for *M. aeruginosa *[13] was employed to genetically characterize the newly isolated strains. Amplified PCR fragments were purified by EXOSAP-IT (USB) and were sequenced in both directions by a DTCS Quick start Kit and a CEQ8000 autosequencer (Beckman Coulter). Following the standard procedure of MLST, each different allele for each locus is assigned a different arbitrary number, and the unique combination of seven allele numbers ("allelic profile") unambiguously defines a strain's sequence type ("ST"). The MLST sequence data of the 164 strains that we previously characterized [13] are available at the DDBJ database [DDBJ:AB324850–325402]. Newly determined MLST sequence data have been deposited in the DDBJ database [DDBJ: AB465739–465899].

### *mcy *multilocus sequencing typing (*mcy*MLST)

Three loci, *mcyD*, *mcyG*, and *mcyJ*, encoding parts of the DH domain (involved in *trans *double-bond formation during the polyketide chain elongation of Adda), the A domain (involved in activation of phenylacetate, the precursor of Adda), and ***o***-methyltransferase (involved in ***o***-methylation of the C_9 _of Adda), respectively, were selected for the multilocus *mcy *gene sequence typing ("*mcy*MLST"). Each locus is expected to be ca. 550 bps in length. Note that these three loci were selected on the basis of three criteria: 1) the domains encoded by these loci are not directly involved in the structural variation of microcystins (at least those identified in this study); 2) the sequences of these loci have diverged sufficiently from other regions of the *mcy *gene cluster to exclude the possible misamplification of similar but different domains by PCR; 3) there is no evidence that these loci have been replaced with the genes of phylogenetically distant organisms through horizontal transmission (as encountered in the A domain of *mcyB1 *[10]). Each locus was PCR-amplified using published primers (DF/DR, GF/GR, and JF/JR) and optimal reaction conditions [12]. Purification and sequencing reactions of the amplified PCR fragments were performed according to the protocol described above for MLST. As in MLST, each different allele for each *mcy*MLST locus was assigned a different arbitrary number, thereby creating an "allelic profile" that unambiguously defined a strain's "*mcy*ST" (Additional file 1). Sequence data of 11 strains that we previously characterized are available at the DDBJ database [DDBJ:AB110114–110146]. Newly determined sequence data have been deposited in the DDBJ database [DDBJ:AB444730–444852].

### Phylogenetic analysis

For phylogenetic analysis, the most appropriate models of the DNA sequence evolution of each *mcy*MLST locus were selected by a hierarchical likelihood ratio test (hLRT) using MODELTEST version 3.7 [17]. Using PAUP* version 4.0b10 [18], neighbor-joining (NJ) phylogenetic trees were constructed on the basis of the maximum-likelihood (ML) distance calculated from the inferred model and parameters. NJ bootstrap (NJBP) analyses were also performed to assess the statistical confidence of nodes on the basis of alignments generated by 1,000 resamplings of the data with the same DNA substitution models used for phylogenetic reconstruction. Bayesian ML phylogenetic reconstruction was performed with Mr. Bayes version 3.1.2 [19]. Using the DNA evolution model chosen by MODELTEST and the NJ tree as a starting tree, two independent runs were performed, each with four chains for 5,000,000–7,500,000 generations (where the convergence diagnostics for each gene hit a stop value of 0.01), in which trees were sampled every 100 generations. Statistical confidence for branch support was assessed by posterior probability (PP) estimated from the 50% majority consensus tree after discarding the burn-in phase of 1,250,000–1,875,000 generations (corresponding to one-fourth of the generation of each run). Phylogenetic analysis of the seven housekeeping genes was performed in the same way as that for *mcy *genes, except that the seven genes were concatenated prior to analysis, and each run was performed for 3,000,000 generations in Bayesian phylogenetic inference (due to computational limitation).

Using ClonalFrame version 1.1 [20], multilocus genealogies and a suite of population genetic parameters to account for the given *mcy *data were inferred. The 50% majority consensus genealogy was generated from the posterior samples of the last 50,000 generations at a thinning interval of 100 after discarding the burn-in phase of first 50,000 generations.

### Population genetic analysis

Estimates of the parameters for DNA sequence divergence, gene diversity *h *= [*n*/(*n*-1)] (1-Σ*p*_*i*_^2^) (where *n *is the number of samples, and *p*_*i *_is the relative frequency of ith allele), nucleotide diversity *π *[21], and a test for neutrality by Tajima's *D *[22] were performed with DnaSP version 4.00 [23]. Coalescent-based estimates of the population recombination rate (*ρ *= 2*Ner*, where *Ne *is the effective population size, and *r *is the recombination rate per locus per generation) and population mutation rate (*θ *= 2*Ne μ*, where *μ *is the mutation rate per locus per generation), minimum numbers of recombination (*R*_*M*_, [24]), and statistical probabilities of likelihood permutation test (LPT, [25]) were calculated by LDhat version 2.1 [25]. Multilocus linkage disequilibrium was assessed by the standardized index of association (*I*_A_^S^, [26]) using the program START version 2 [27]. *I*_A_^S ^is a standardized measure of *I*_A _[28], ranging from 0 (panmixia) to 1 (absolute linkage disequilibrium). The statistical significance of non-zero values of *I*_A_^S ^was inferred on the basis of a comparison of those values estimated from 1,000 randomized datasets under a null hypothesis of panmixia. The phi (*Φ*)-test (a robust statistical test for recombination [29]) was performed with Splitstree version 4.8 [30]. Maximum likelihood-based tests for phylogenetic congruence between loci [31] were performed with PAUP*. Analysis of molecular variance (AMOVA) was performed with ARLEQUIN version 3.1 [32]. The McDonald-Kreitman test [33] was performed with DNASP. PAML version 3.14 [34] was used to search for sites under positive selection. Possible positively selected sites were also investigated on the basis of 516 physicochemical criteria with TreeSAAP version 3.2 [35], following the standard protocol except that the statistical significance level was set to 0.001 (*P *< 0.001).

### Microcystin analysis

To a 10-ml culture of each *M. aeruginosa *strain, 0.5 ml of acetic acid was added and the mixture was ultrasonicated for 15 minutes. After centrifugation (3,000 rpm, 15 min), the supernatant was collected and the remnant pellet was further extracted with 1.5 ml of methyl alcohol by the same procedure as the initial extraction. To the combined supernatant, distilled water was added to 20 ml. The diluted extract was passed through a conditioned (1 ml of 100% methyl alcohol and 1 ml of distilled water) Inertsep RP-1 cartridge (GL Science). The cartridge was washed with 20% methanol aqueous solution, and then eluted with 0.5 ml of 80% methanol aqueous solution. The eluate was diluted with 0.5 ml of distilled water, and applied to high-performance liquid chromatography (HPLC) using an LC-10A system (Shimadzu; column: Agilent Eclipse XDB RP-18, 2.1 × 150 mm; solvent: 60% methanol in 50 mM phosphate buffer, pH 3.0; flow rate: 0.2 ml/min; detection: photodiode array detector). Microcystin variants were identified in comparison with authentic samples in the case of microcystins -LR, -YR, and -RR. Other microcystin variants were identified by liquid chromatography electrospray ionization mass spectrometry (LC ESI-MS) using an LCMS-2010A system (Shimadzu).

## Results

### Phylogenetic analysis of the seven housekeeping genes

**Figure 2:**
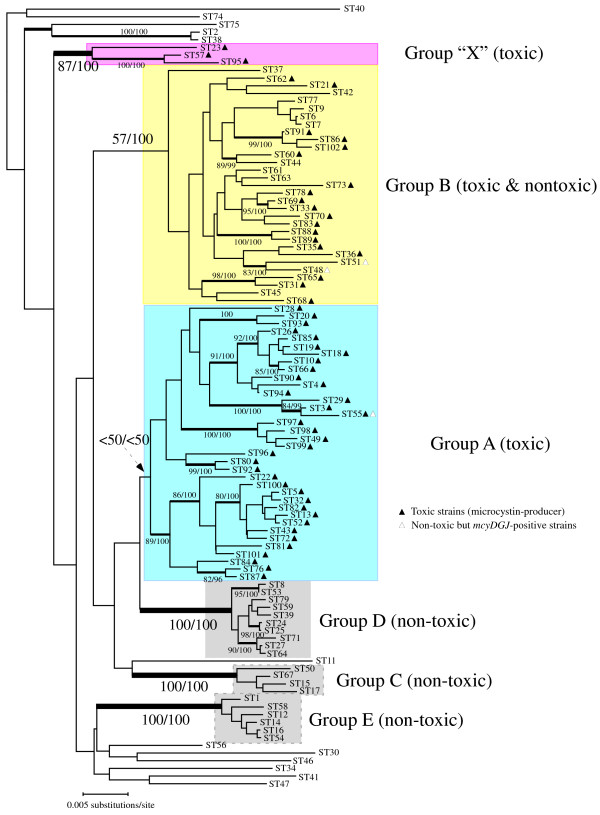
**Phylogenetic analysis of the seven housekeeping genes (MLST)**. Neighbor-joining (NJ) phylogenetic tree of 102 STs of *M. aeruginosa *based on the concatenated sequences of the seven MLST loci. Branches supported by both NJ bootstrap probabilities (NJBP > 75%) and Bayesian posterior probabilities (PP > 0.85) are highlighted by thick bars. Statistical values for these branches are indicated (NJ BP, PPx100).

The results of the phylogenetic analysis of the seven housekeeping (MLST) loci is shown in Fig. 2. Overall, the results were consistent with those of our previous study [13]. The most toxic strains fell into two lineages, termed group A and group B. The statistical support for group A was weak, whereas that for group B was moderate to strong. In group A, all but one strain represented by ST55 were toxic (microcystin-producer), whereas toxic and non-toxic strains coexisted in group B. Three toxic strains represented by ST23, ST57 and ST95 belonged to neither group A nor group B, forming a distinct monophyletic lineage (designated group "X"). The location of ST40 was ambiguous and varied according to the phylogenetic method used, a feature that was also encountered in our previous study [13]. Three distinct non-toxic clades (groups C, D, and E) were again recovered in this phylogenetic analysis.

### *mcy *multilocus sequence typing (*mcy*MLST)

The results of the *mcy*MLST analysis are shown in Additional file 1. All sets of primers successfully recovered the three *mcy *loci from all toxic and *mcyG*-positive isolates included in this study. By contrast, the primer sets for *mcyD *and *mcyJ *failed to amplify these genes from any of the *mcyG*-negative strains that we identified in our previous study [13]. The amplified loci of *mcyD*, *mcyG*, and *mcyJ *were 550, 449, and 552 bp, respectively, in length. Neither insertions nor deletions were found within the sequences of *mcyG *and *mcyJ*, whereas an insertion of 3 bp was found within one *mcyD *genotype (allele number 18 of *mcyD *in *mcy*ST25) representing five strains. All sequences could be unambiguously aligned (note that the 3-bp insertion in allele 18 of *mcyD *is TTT, which might be a slippage mutation of the prior TTT sequence). For the 118 *mcy*-positive isolates, 51 *mcy *sequence types (*mcy*STs) were found. The number of alleles of *mcyD*, *mcyG*, and *mcyJ *was 27, 31, and 29, respectively.

### Phylogenetic analysis of *mcy*

**Figure 3:**
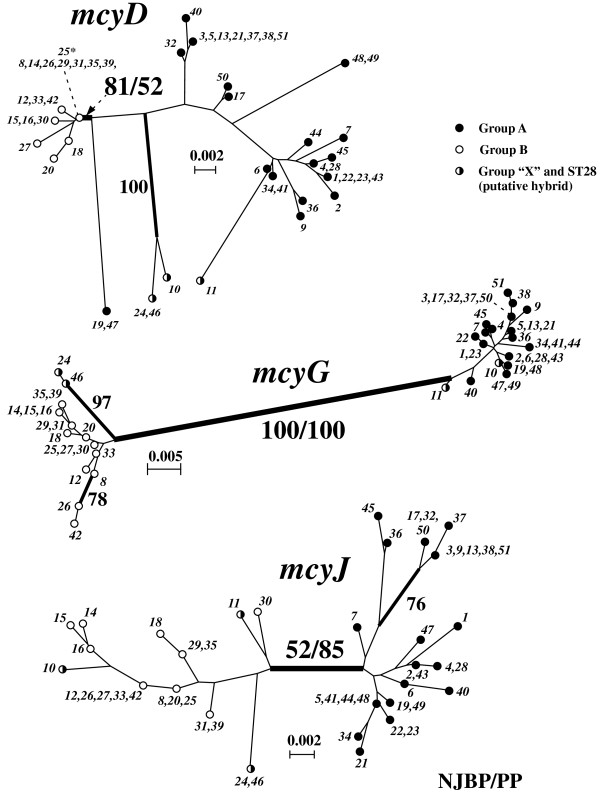
**Neighbor-joining tree of *mcy *genes**. Phylogenetic relationships among the 51 *mcy*STs of *M. aeruginosa *based on the NJ analysis of the individual *mcy *loci. Statistical values for the branch of the group A-B boundary (in bold) are indicated (NJ bootstrap [NJBP]/[Bayesian PP] × 100). In general, within-group relationships were poorly resolved; branches with NJBP > 75% are highlighted in bold and NJBP values are shown.

**Figure 4:**
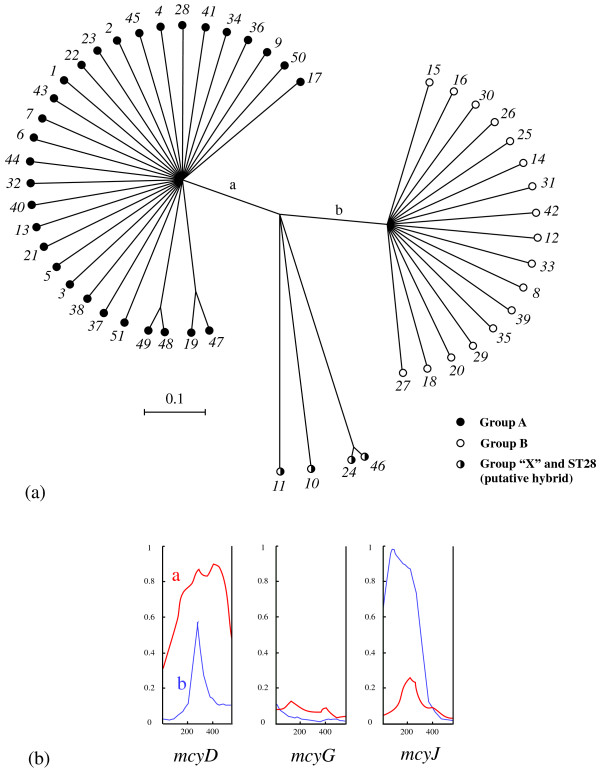
**ClonalFrame analysis of *mcy *genes**. (a) A ClonalFrame genealogy of the 51 *mcy*STs inferred from the three *mcy *loci. (b) Genetic representation of the recombination events at the branch of a (red) and b (blue) in the *mcy *genealogy. X-axis indicates the nucleotide position in the respective *mcy *loci. Y-axis indicates the posterior probability of recombination.

The results of the phylogenetic analysis of the individual *mcy *loci is shown in Fig. 3. In all of the three *mcy *phylogenetic trees, *M. aeruginosa *strains could be separated into two clades, group A and group B, consistent with the same grouping as in the MLST phylogeny (Fig. 2). This dichotomy was most robustly supported in the phylogenetic tree of *mcyG *(NJ BP100%, Bayesian PP 100%), whereas it was moderately supported in the *mcyD *and *mcyJ *phylogenies. Four *mcy*STs (*mcy*ST10, *mcy*ST11, *mcy*ST24, and *mcy*ST46) showed discordant placements between the two groups (A and B) depending on the loci used. For example, *mcy*ST10 was located within group A by *mcyD *and *mcyG *analysis, but within group B by *mcyJ *analysis. Such discordance is highly likely to be due to recombination between loci. Therefore we next employed ClonalFrame, a multilocus phylogenetic reconstruction method that takes into account the effect of recombination between and within loci. The results of ClonalFrame, which showed the highest likelihood value of 10 independent MCMC runs, are illustrated in Fig. 4a. Again, two highly supported clades (Bayesian PP > 95%) were recovered. Four anomalous *mcy*STs (*mcy*ST10, *mcy*ST11, *mcy*ST24, and *mcy*ST46) identified in the individual *mcy *phylogenies branched at the midpoint of groups A and B. Within-group relationships were still poorly resolved, as in the analyses of individual loci.

### Genetic diversity and recombination

Because two distinct groups were identified in the *mcy *phylogenetic and genealogical analyses, genetic diversity indices were estimated from all of the *mcy *data and also from subsets of data (groups A and B) (Table 1). The maximum sequence divergence of *mcyD*, *mcyG*, and *mcyJ *in the whole dataset was 3.6%, 5.1%, and 2.9%, that in group A was 3.6%, 1.5%, and 2.0%, and that in group B was 0.7%, 2.0%, and 2.0%, respectively. For *mcyD*, the genetic diversity differed between groups, being much higher in group A (*S *= 37, *π *= 0.0142) than in group B (*S *= 6, *π *= 0.0017). For *mcyG *and *mcyJ*, the genetic diversity indices were similar between the groups.

**Table 1:** Statistical tests and parameter estimates for mutation and recombination

		***S*^1^**	***π*^2^**	***D*^3^**	***ρ*^4^**	***θ*^5^**	***ρ/θ***	***R*_*M*_^6^**	***P*_*LPT*_^7^**	***P*_*Φ*_^8^**
***mcyD***	**All (*n *= 118)**	43	0.01529	0.133	12.6	8.047	1.56	10	0.075	0.6829
	**Group A^9 ^(*n *= 89)**	37	0.01427	0.230	14.62	7.114	2.05	10	0.037*	0.875
	**Group B^9 ^(*n *= 29)**	6	0.00171	-1.105	4.97	1.528	3.25	0	0.020*	1.00
***mcyG***	**All (*n *= 118)**	39	0.0193	1.402	1.7	6.176	0.275	5	0.298	0.5092
	**Group A^9 ^(*n *= 87)**	14	0.00559	0.289	10.95	2.382	4.59	2	0.018*	0.012*
	**Group B^9 ^(*n *= 31)**	19	0.00635	-0.921	0	4.506	0	0	0.08	1.00
***mcyJ***	**All (*n *= 118)**	27	0.0128	1.174	19.5	5.053	3.85	7	0.012*	0.0012**
	**Group A^9 ^(*n *= 84)**	16	0.00895	1.559	17.6	3.199	5.5	5	0.034*	0.026*
	**Group B^9 ^(*n *= 34)**	18	0.00712	-0.359	18.4	4.402	4.17	4	0.362	0.428

* *P *< 0.05, ** *P *< 0.01

^1 ^Number of segregating sites.

^2 ^Nucleotide diversity [21].

^3 ^Tajima's *D *[22].

^4 ^Population recombination rate (= *2Ner*) estimated from the sites with two alleles.

^5 ^Population mutation rate (= 2*Neμ*) estimated from the sites with two alleles.

^6 ^Minimum number of recombination based on Hudson and Kaplan [24].

^7^*P*-value of likelihood permutation test [25].

^8^*P*-value of *Φ *test [29].

^9^STs that show discordant group assignment between loci (ST10, 11, 24, and 46) are also included.

Two statistical tests, the likelihood permutation test (LPT) and phi (*Φ*) test were performed to explore recombination within loci (Table 1). All tests for intragenic recombination within group A were significant except for one case (*Φ *test of *mcyD*), whereas recombination within group B was not significant in any tests except for the LPT of *mcyD*. Consistent with these results, the minimum number of recombination (*R*_*M*_) was larger in group A than in group B, and *mcyD *and *mcyG *of group B showed no evidence of recombination (*R*_*M *_= 0). Coalescent-based parameter estimates indicated that the population recombination rates (*ρ*) were generally larger than population mutation rates (*θ*). Once again, *ρ *values for *mcyD *and *mcyG *were higher in group A than in group B, but similar between the groups for *mcyJ*. On the other hand, statistical tests using the standardized index of association (*I*_*A*_^S^) for all samples, group A, and group B gave significant positive values (*P *< 0.01) (Table 2). However, *I*_*A*_^S ^using only unique STs gave significant positive values for the whole dataset and group A (*P *< 0.01), whereas the *I*_*A*_^S ^of group B was not significantly different from zero (*P *= 0.167). Maximum likelihood-based tests for phylogenetic congruence demonstrated (Table 2) that significant congruence (*P *< 0.01) between the *mcy *ML trees and randomized trees was present for the whole dataset and group A, but not for group B, indicating that recombination is much more frequent in group B. Overall, the results were consistent but gave different effects of recombination between groups; that is, intralocus recombination is frequent within group A but rare within group B, whereas interlocus recombination is frequent within group B but not within group A. Interestingly, the mutation-scaled recombination rate (*ρ/θ*) of *mcyG *was 20 times larger for group A than for the whole dataset (Table 1). Recombination at a specific branch in the phylogenetic tree can be detected by the program ClonalFrame. For example, our analysis indicated that small segments of *mcyD *and *mcyJ *are likely to have undergone recombination at the branch of the group A-B boundary (Fig. 4b).

**Table 2:** Analyses of interlocus recombination

		***n*^1^**	***I*_*A*_^*S*2^**	**Portion of significant congruence^3^**
**Total**	**All**	118	0.656*	
	**ST**	51	0.188*	6/6
**Group A**	**All**	84	0.680*	
	**ST**	30	0.193*	6/6
**Group B**	**All**	29	0.359*	
	**ST**	17	0.064	0/6

^1 ^Number of strains.

^2 ^Standardized index of association [26]. *, *P *< 0.01.

^3 ^Maximum likelihood analysis of tree topology congruence based on Feil *et al*. [31]. The log-likelihoods of 200 random trees were calculated based on the sequence data of each locus, and compared to those of the ML tree topologies of the other two loci. Log likelihood scores higher than those of 99th percentile of 200 random tree topologies are "significantly congruent" (*P *< 0.01), suggesting the low rate or lack of recombination. Thus, the lower value of the portion of significant congruence indicates the higher rate of recombination.

### Test for selection

The inferred parameter values indicative of selection and the results of tests for selection are shown in Table 3. The ratio of nonsynonymous changes per nonsynonymous site (*d*_N_) to synonymous changes per synonymous site (*d*_S_) may provide a good measure of adaptive evolution on a given locus because an excess of *d*_N _over *d*_S _(i.e., *d*_N_/*d*_S _>1) is expected under adaptive evolution. However, the *d*_N_/*d*_S _values of *mcyD*, *mcyG*, and *mcyJ *were 0.227, 0.243, and 0.169, respectively, values that are all much smaller than 1, indicating that purifying selection rather than positive selection is responsible for the genetic diversity of the *mcy *genes.

**Table 3:** Test for selection

	***d*_N_/*d*_S_^1^**	***P*_MK_^2^**	***P*_PAML_^3^**	**n_M_^4^**	**n_M _(A-B)^5^**	**n_TS_^6^**	**n_TS _(A-B)^7^**
***mcyD***	0.2270	> 0.50	> 0.50	0	0	24	1
***mcyG***	0.2437	> 0.50	< 0.001	2 (M2a)	2	15	7
***mcyJ***	0.1696	> 0.50	< 0.001	2 (M8)	1	10	1

^1^Ratio of nonsynonymous substitutions per nonsynonymous sites to synonymous substitutions per synonymous sites based on M0 model of PAML.

^2^*P*-value of the McDonald-Kreitman test (group A vs. B) [33].

^3^*P*-values of likelihood ratio tests for both M1a vs. M2a, and M7 vs. M8 in PAML [34]. Significant value indicates the presence of positively selected sites.

^4 ^Number of codons under positive selection detected by the model showing the highest likelihood (indicated in parentheses) of PAML (*P *< 0.05).

^5 ^Number of codons under positive selection changed on the branch of the group A-B boundary according to PAML using the model described in n_M _column.

^6 ^Number of codons under positive selection according to TreeSAAP [35].

^7 ^Number of codons under positive selection changed on the branch of the group A-B boundary according to TreeSAAP.

Next, we performed several statistical tests to investigate further the presence of selection acting on *mcy *genes. The Tajima's *D *statistic indicates selection when it significantly differs from the neutral expectation of *D *= 0. As shown in Table 1, the Tajima's *D *value for each *mcy *locus did not significantly differ from zero for the whole dataset or group A or B (*P *> 0.10). To investigate whether selection was responsible for the deep divergence of *mcy *into groups A and B, we performed the McDonald-Kreitman (MK) test, which compares the ratio of synonymous to non-synonymous polymorphism within groups and that of synonymous to non-synonymous divergence (fixed differences) between groups; these two ratios should be equal under the null hypothesis of neutrality [33]. Again, none of the MK test *P *values was significant (Table 3), and thus did not provide evidence for selection on the branch of the group A-B boundary.

All of the above analyses are based on the average nucleotide polymorphism; however, these kinds of test are not sensitive enough to detect a single or small number of adaptive amino acid changes, which are sometimes responsible for improved fitness of a given gene. Two programs, PAML and TreeSAAP, were therefore used to overcome this problem. The first program PAML uses the same approach as the *d*_N_/*d*_S _consideration but applies it to individual sites on the basis of maximum-likelihood, thus enabling codons under positive selection to be detected. Using PAML, we employed the "site models", and performed two likelihood-ratio tests, a test of M1a (nearly neutral) versus M2a (positive selection), and that of M7 (beta; assuming a beta distribution of *d*_N_/*d*_S _over sites ranging from 0 to 1) versus M8 (beta & ω; the same as M7 with an additional estimate of ω = *d*_N_/*d*_S _> 1) to determine any sites under positive selection (*d*_N_/*d*_S _> 1), as suggested by the author [34]. Significant likelihood ratio values were obtained for *mcyG *and *mcyJ *(*P *< 0.01), but not for *mcyD *(*P *> 0.50). For each locus (*mcyG *and *mcyJ*), two codons were identified as positively selected sites by the model that showed the highest log-likelihood (the M2a model for *mcyG*, and M8 model for *mcyJ*). Although the number was small (up to two), the most likely positively selected sites were substituted on the branch bordering groups A and B.

The second program, TreeSAAP, provides a method to detect an adaptive amino acid change by taking quantitative amino acid properties into account. On the basis of statistical tests for differences in the observed versus the expected change under neutrality in the eight categories of 516 physicochemical criteria (e.g., changes in hydropathy, isoelectric point, polarity, and so on), TreeSAAP identified 24, 15, and 10 sites under positive selection in *mcyD*, *mcyG*, and *mcyJ*, respectively; these numbers of sites were much larger than those identified by PAML. TreeSAAP also identified all of the positively selected sites identified by PAML, including the sites at the branch of the A-B boundary. Unlike the result of PAML analyses, the most likely positively selected sites identified in *mcyD *and *mcyJ *did not change on the branch bordering groups A and B, whereas half of the selected sites in *mcyG *were identified to have been substituted on the branch of the A-B boundary.

### Genetic differentiation

To investigate the significance of geographic isolation on the genetic diversity of the *mcy *genes, our strains were partitioned into their geographic origins. Because the inclusion of localities with a small number of strains may bias the result of AMOVA, we included only strains for which more than five isolates were available from a single locality. On the basis of this criterion, nine groups of strains from Lake Barato (*n *= 6), Lake Inba (*n *= 22), Lake Kasumigaura (*n *= 22), Lake Okutama (*n *= 12), Lake Suwa (*n *= 5), Lake Teganuma (*n *= 11), Ishigaki Dam (*n *= 5) and Kunnma Dam (*n *= 6) were selected and analyzed. The results of AMOVA (Table 4) indicated low but significant genetic structuring among local populations (*F*_ST _= 0.212, *P *< 0.001), although the within-population genetic variance was still high.

**Table 4:** Analysis of molecular variance (AMOVA)

**Source of variation**	**d.f**.	**Sum of squares**	**Variance component**	**% of variation**	***F*_ST_**	***P *value**
**Among local populations**	8	282.739	2.83773	21.26		
**Within populations**	73	767.395	10.51226	78.74		

**Total**	81	1050.134	13.34999		0.212	< 0.001

### Microcystin analysis

**Figure 5:**
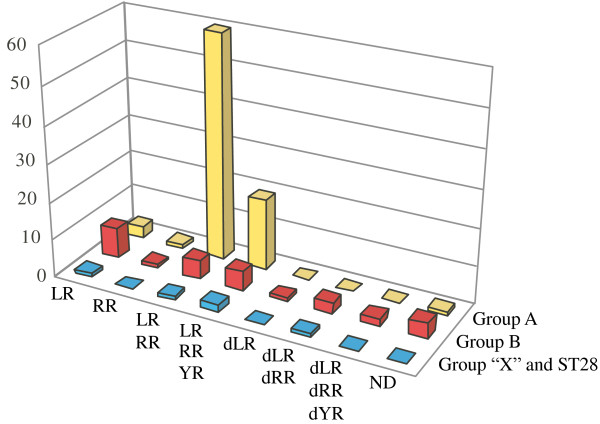
**Microcystin composition**. Distribution of microcystin variants in groups A, B, and ''X'' (putative hybrid *mcy*STs: *mcy*ST10, *mcy*ST11, *mcy*ST24, and *mcy*ST46). Abbreviations for the microcystin variants are as follows: LR, microcystin-LR; RR, microcystin-RR; YR, microcystin-YR; dLR, [Dha^7^]microcystin-LR; dRR, [Dha^7^]microcystin-RR; dYR, [Dha^7^]microcystin-YR; ND, not detected.

Most strains were found to produce multiple variants of microcystins (Additional file 1). On the basis of the microcystin composition, our strains were divided into eight categories (Fig. 5). Several isolates were found to produce other microcystin variants at a low concentration (less than 10% of the total amount of microcystins). We did not include them here, because such minor variants are highly likely to have been produced through the occasional misrecognition of amino acids by the substrate-binding pocket of the A domain and therefore would appear to be not selectively important.

The most dominant form of microcystin was microcystin-LR and -RR, representing 56% of the total 118 *mcy*-positive isolates. The second most dominant form was microcystin-LR, -RR, and -YR, which accounted for 22% of the 118 strains. In general, group B appeared to be more divergent in microcystin composition as compared with group A. For example, demethylated microcystin variants ([Dha^7^]microcystin-LR, -RR, and -YR, Fig. 1) were exclusively present in strains assigned to group B (except for the anomalous *mcy*ST10), although genotypic relationships among the different variants were poorly resolved. Indeed, the difference in microcystin composition between group A and group B was significant (*χ*^2 ^= 44.7, d.f. = 6, *P *< 0.001). Some strains with the same *mcy*ST were found to produce a different combination of microcystin variants (e.g., *mcy*ST1, see Additional file 1). Once again, it should be noted here that five *mcy*-positive isolates did not produce a detectable level of microcystins [13] (Additional file 1).

## Discussion

Phylogenetic analysis based on the seven housekeeping loci (MLST) indicated that toxic strains are affiliated into two major clades, group A and group B, and an outlier clade group "X" (Fig. 2). This observed subgrouping of toxic strains is largely consistent with our previous MLST phylogeny [13], suggesting that the ability to produce microcystins is genetically structured to some extent. On the other hand, our phylogenetic and multilocus genealogical analyses of the *mcy*MLST divided our strains into two clades (Fig. 3, 4a). Importantly, these two clades correspond to groups A and B in the MLST phylogeny. The congruent phylogenies between the *mcy *(*mcyA*, *mcyD*, *mcyG*, and *mcyE*) and housekeeping genes (e.g., 16S rRNA) of several microcystin-producing cyanobacterial genera have been demonstrated and suggested to be evidence for the vertical rather than interspecific horizontal transmission of *mcy *genes [10,36]. The overall concordance of group assignment between the *mcy *and MLST phylogenies in this study suggests that horizontal transmission of *mcy *between distantly related *Microcystis *strains is also not so frequent. Interestingly however, strains included in group "X" and ST28 (*mcy*ST11) in group A show discordant phylogenetic placements between groups A and B in the *mcy *phylogeny depending on the *mcy *loci used, suggesting that these strains represent the consequence of intergroup horizontal transmissions of *mcy *genes followed by recombination.

Our population genetic data corroborate our previous finding that recombination is an important force in maintaining the genetic diversity of the *mcy *genes [12]. Moreover, population recombination rates were larger than mutation rates in general (*ρ/θ *>1, Table 1), suggesting that recombination is more frequent than point mutation within the *mcy *genes. Although our data may not be robust owing to the small sample sizes, the relative contribution of recombination and point mutation to the genetic diversity of the *mcy *genes may be comparable to that of various genes of other highly recombinogenic bacteria, such as *Helicobacter pylori *and *Streptococcus pneumoniae *[37]. ClonalFrame assumes that recombined fragments (imported donor sequence) differ from the parental segments (original recipient sequence) at a constant percentage of ν, which can be used to roughly infer the origin of recombined fragments [20]. Our estimate of ν of *mcy *is 0.018, which is lower than the maximum sequence divergence (0.050) of *mcy *genes. This result suggests that most recombined segments originated from within *M. aeruginosa*. ClonalFrame also estimated the mean length of recombined segments as 206 bp, which is in line with a previous study showing that bacterial recombination can occur within a very short length of DNA (<1 kbp) [38]. The very small size of the imported segments implies that they are subject to various restriction systems [39] discovered within the genome of *M. aeruginosa *[8].

Within clades, recombination was suggested to be frequent, although the effect of recombination appeared to differ between groups and loci. For example, interlocus recombination in group B was so frequent that alleles at different *mcy *loci were assorted randomly ("panmictic"), whereas the clonal divergence of *mcy *genes was suggested in group A (Table 2). When we examined intralocus recombination, however, most significant recombination was found for group A, with little recombination for group B (Table 1). The different impact of recombination between two clades might be due to the different forms of genetic exchange and restriction system, because the length of recombined segments is known to be highly dependent on these mechanisms [40].

Importantly, statistical tests indicated that recombination seems to be more substantial within groups than between (Table 1, 2), implying that there is genetic isolation between groups A and B owing to DNA sequence divergence. This result suggests that homology-dependent recombination makes a significant contribution to the strong genetic clustering of *mcy *genes. In this context, each *mcy *group may be recognized as a fuzzy analog of "biological species" in higher eukaryotes [41]. Accordingly, strains belonging to group "X" might represent "hybrids" that could successfully cross over the genetic barrier between the two groups A and B. Bacterial recombination is often mediated by vectors (e.g., phages). Some cyanophages that are infectious to *Microcystis *have been suggested to have a very narrow host range [42]. Such vector specificity might play a role in augmenting the genetic isolation between the two *mcy *clades. Finally, the hypothesis that recombination might also play an important role in the diversification of *mcy *genes should not be dismissed. As shown in Fig. 4b, significant recombination is likely to have occurred on the branch bordering groups A and B.

Our data suggest that selection has not been an important factor in the genetic divergence of the *mcy *genes. None of Tajima's *D*-values was significantly different from the neutral assumption, and the MK test failed to reject the neutral divergence of *mcy *into the two groups A and B. In addition, the PAML and TreeSAAP analyses identified few positively selected sites within the *mcy *genes on the branch separating groups A and B. One exception was the TreeSAAP analysis of *mcyG*, which identified seven possible sites under positive selection on the branch of the group A-B boundary. The product of *mcyG *is probably involved in polyketide chain elongation within the Adda of microcystins [5]. All microcystin variants identified in this study share the same Adda at the corresponding position of the microcystin (Fig. 1), and therefore positive selection at these sites, if present, appears to have little significance with regard to microcystin structure. It is possible that the observed deep divergence of *mcyG *arose as a result of selection at different but linked *mcy *genes. If so, it would be expected that these two clades would differ in microcystin composition, which might be thought to confer selective advantages under different (but unknown) ecological conditions. However, analysis of microcystin composition did not support this hypothesis. Although demethylated types of structural microcystin variants are exclusively present in strains in group B (except for *mcy*ST10 in group "X"), the most frequently found microcystin compositions (e.g., microcystin-LR and -RR, and microcystin-LR, -RR, and -YR) are present in both groups (Fig. 5), suggesting that *mcy *genealogy is decoupled from microcystin variation. Similar results have been obtained for the freshwater cyanobacterium *Planktothrix*, in which discordance between the genetic relationships and compositional trends of nonribosomal peptides is encountered [43]. Moreover, it has been demonstrated that positive selection at the amino acid site neighboring the binding pocket of the A domain of *mcyB *in *Microcystis *does not contribute to the production of specific microcystin variants [11]. Unfortunately, the biological role of microcystins has yet to be determined, although several possible functions have been proposed [44-46]. Clarifying the function of microcystins will be critical to evaluate further the importance of selection on the *mcy *genes.

As described above, strains affiliated to groups A and B in the MLST phylogeny (Fig. 2) are generally clustered in the same groups in the *mcy *phylogeny (Fig. 3, 4a). We previously suggested that the intraspecies clades found in the MLST phylogeny might represent "ecotypes" [13]. In this context, it is possible that selective sweeps on linked loci outside the *mcy *genes yielded the structured *mcy *phylogeny (i.e. a "hitchhiking effect"). For *M. aeruginosa*, several morphological (e.g., colony morphology [47]) and physiological (e.g., photosynthetic pigments [48]) variations are known. As shown in other cyanobacteria [49], positive selection may favor these characteristics, but none of them is specific to either of the two groups (A and B) in *M. aeruginosa *(YT, unpublished data). Of course, investigation of previously uncharacterized ecological parameters are needed to rule out the possibility of *mcy *divergence due to a hitchhiking effect. In any event, selection acting on the *mcy *genes has little impact on their deep divergence with regard to the variation of microcystin composition.

Genetic clustering can arise in the absence of selection where gene flow among populations is restricted due to a geographic barrier. Although a few exceptions are known [50], this possibility has been considered unlikely for microbes including *M. aeruginosa *for which long-distance dispersal may be easy owing to their small cell size and immense population size [51]. In fact, *M. aeruginosa *strains belonging to different groups in the *mcy *phylogenies are often isolated from a small amount of water sampled from one location at one time (Additional file 1), suggesting that allopatry is not an important factor in the genetic isolation observed between the two *mcy *groups. Consistent with this observation, the results of our AMOVA found little geographic contribution to the pattern of genetic variation within the *mcy *genes (Table 4).

Despite the absence of geographic isolation and selection, we have identified two distinct *mcy *clusters for which recombination is much more frequent within than between. These results suggest that recombination and neutral genetic drift are primarily responsible for the observed deep divergence of the *mcy *genes in *M. aeruginosa*. This pattern of bacterial genetic divergence is in line with recent theoretical results indicating that genotypic clusters can arise and be maintained in the absence of selection or physical isolation when the recombination rate is a negative log-linear function of genetic distance [52,53] and the effective population size (*Ne*) is extremely large [54]. Although the *Ne *of a bacterial population is difficult to appreciate and would be at least much lower than the census population size [53], *M. aeruginosa *often forms a bloom with extremely large numbers of cells (sometimes exceeding 10^6 ^cell/ml [55]) with a high level of neutral genetic diversity [13], and is thus likely to fulfill the latter condition [54]. Recently, neutral molecular evolution in the natural bacterial population has received more attention than previously, and indeed potential evidence for it has been reported [56,57].

## Conclusion

Our phylogenetic and population genetic analyses of multiple conservative loci within the microcystin synthetase (*mcy*) gene cluster suggested that *mcy *genes of *M. aeruginosa *are subdivided into two cryptic clades, which have been primarily generated and maintained as a result of homology-dependent recombination and neutral genetic drift.

## Abbreviations

AMOVA: analysis of molecular variance; BP: bootstrap probability; MLST: multilocus sequence typing; NJ: neighbor-joining; PP: posterior probability; ST: sequence type.

## Authors' contributions

YT designed the research, YT and MMW collected samples, YT performed isolation, culturing, DNA experiments, phylogenetic and population genetic analyses, YT and TS performed microcystin analyses, YT, TS, and MMW wrote the paper, and YT, FK, and MMW coordinated the research. All authors read and approved the final manuscript.

## Supplementary Material

Additional File 1 **Strain information**. Genotypic and toxin profiles of strains used in this study. Click here for file
