# Supplementary material for: Recombination, cryptic clades and neutral molecular divergence of the microcystin synthetase (mcy) genes of toxic cyanobacterium Microcystis aeruginosa
Source: BMC Evol Biol. 2009 May 22;9:115. doi: 10.1186/1471-2148-9-115 (PMC2693435; doi:10.1186/1471-2148-9-115)
Supplement: Additional File 1 — Strain information. Genotypic and toxin profiles of strains used in this study. [file 1471-2148-9-115-S1.pdf]

Additional file 1. Strain information.

Strain	Locality[1]	Year	MLST		Group[2]	*mcy* MLST		Microcystin[3]					
			ST[4]	AP[5]		*m* ST[6]	AP[7]	LR	RR	YR	dLR	dRR	dYR
NIES88	Lake Kawaguchi	1981	**3**	3,3,3,3,3,3,3	A	***1***	1,1,1	+	+	-	-	-	-
TAC60	Lake Shirakaba	1982	**29**	18,21,3,17,3,14,21	A	***1***	1,1,1	+	+	+	-	-	-
TAC61	Lake Shirakaba	1982	**29**	18,21,3,17,3,14,21	A	***1***	1,1,1	+	+	+	-	-	-
TAC97	Lake Shirakaba	1984	**29**	18,21,3,17,3,14,21	A	***1***	1,1,1	+	-	-	-	-	-
TAC175	Lake Shirakaba	1989	**29**	18,21,3,17,3,14,21	A	***1***	1,1,1	+	+	-	-	-	-
TAC176	Lake Shirakaba	1989	**29**	18,21,3,17,3,14,21	A	***1***	1,1,1	+	+	-	-	-	-
TAC183	Lake Shirakaba	1989	**29**	18,21,3,17,3,14,21	A	***1***	1,1,1	+	+	-	-	-	-
TAC182	Lake Teganuma	1989	**29**	18,21,3,17,3,14,21	A	***1***	1,1,1	+	+	-	-	-	-
TAC71	Hakui, Isikawa	1982	**4**	4,4,4,4,4,4,4	A	***2***	2,2,2	+	+	-	-	-	-
NIES89	Lake Kawaguchi	1981	**4**	4,4,4,4,4,4,4	A	***2***	2,2,2	+	+	-	-	-	-
TAC62	Lake Kutsuzawa	1982	**4**	4,4,4,4,4,4,4	A	***2***	2,2,2	+	+	-	-	-	-
TAC63	Lake Kutsuzawa	1982	**4**	4,4,4,4,4,4,4	A	***2***	2,2,2	+	+	+	-	-	-
TAC166	Lake Okutama	1989	**4**	4,4,4,4,4,4,4	A	***2***	2,2,2	+	+	-	-	-	-
Tn05AK02	Lake Teganuma	2005	**94**	43,4,66,43,4,8,4	A	***2***	2,2,2	+	+	+	-	-	-
NIES90	Lake Kawaguchi	1981	**5**	5,5,5,5,5,5,5	A	***3***	3,3,3	+	+	-	-	-	-
NIES103	Lake Kasumigaura	1978	**10**	9,9,8,8,9,8,8	A	***4***	4,4,4	+	+	-	-	-	-
NIES102	Lake Kasumigaura	1982	**10**	9,9,8,8,9,8,8	A	***4***	4,4,4	+	+	+	-	-	-
NIES107	Lake Kawaguchi	1981	**13**	5,5,5,10,5,11,10	A	***5***	3,5,5	+	+	+	-	-	-
NIES843[T 8]	Lake Kasumigaura	1997	**18**	9,15,15,12,16,14,13	A	***6***	5,2,6	+	+	+	-	-	-
CL4	China	1996	**19**	9,15,15,12,16,14,8	A	***6***	5,2,6	+	+	-	-	-	-
NIES903	China	1996	**26**	9,15,21,12,16,8,8	A	***6***	5,2,6	-	+	-	-	-	-
TAC92	Lake Barato	1984	**26**	9,15,21,12,16,8,8	A	***6***	5,2,6	+	+	+	-	-	-
TAC93	Lake Barato	1984	**26**	9,15,21,12,16,8,8	A	***6***	5,2,6	+	+	-	-	-	-
TAC38	Lake Kasumigaura	1978	**26**	9,15,21,12,16,8,8	A	***6***	5,2,6	+	+	-	-	-	-
TAC45	Lake Kasumigaura	1978	**26**	9,15,21,12,16,8,8	A	***6***	5,2,6	+	+	-	-	-	-
TAC46	Lake Kasumigaura	1978	**26**	9,15,21,12,16,8,8	A	***6***	5,2,6	+	+	+	-	-	-
Ks05TA61	Lake Kasumigaura	2005	**26**	9,15,21,12,16,8,8	A	***6***	5,2,6	+	+	-	-	-	-
Kv05YA06	Lake Kitaura	2005	**26**	9,15,21,12,16,8,8	A	***6***	5,2,6	+	+	-	-	-	-
TAC162	Lake Teganuma	1989	**26**	9,15,21,12,16,8,8	A	***6***	5,2,6	+	+	+	-	-	-
Ks05YA05	Lake Kitaura	2005	**85**	9,15,15,46,16,8,8	A	***6***	5,2,6	+	+	-	-	-	-
Sw5	Lake Suwa	2004	**85**	9,15,15,46,16,8,8	A	***6***	5,2,6	+	+	-	-	-	-
MCS3	Thailand	2000	**20**	13,16,16,13,17,3,14	A	***7***	6,6,7	+	-	-	-	-	-
TL2	Thailand	1997	**22**	15,18,18,5,19,11,16	A	***9***	8,8,3	+	+	-	-	-	-
TAC86	Koshi-ike	1984	**28**	17,20,23,16,23,18,20	A	***11***†	10,10,10	+	+	+	-	-	-
TAC109	Shigure Dam	1984	**28**	17,20,23,16,23,18,20	A	***11***†	10,10,10	+	+	+	-	-	-
TAC350	Lake Toro	1990	**32**	5,24,5,10,5,21,5	A	***13***	3,5,3	+	+	-	-	-	-
TAC129	Kamisawa-ike	1989	**43**	5,5,34,26,5,28,30	A	***17***	13,3,15	+	+	-	-	-	-
TAC177	Lake Shirakaba	1989	**49**	30,15,23,31,37,34,34	A	***19***	15,14,17	+	+	-	-	-	-
TAC178	Lake Shirakaba	1989	**49**	30,15,23,31,37,34,34	A	***19***	15,14,17	+	+	-	-	-	-
TAC135	Lake Suwa	1989	**52**	5,5,5,10,5,11,36	A	***21***	3,5,18	+	+	+	-	-	-
TAC165	Lake Okutama	1989	**55**	31,41,3,3,3,35,37	A	***22****	1,16,19	-	-	-	-	-	-
TAC167	Lake Okutama	1989	**55**	31,41,3,3,3,35,37	A	***23***	1,1,19	+	+	-	-	-	-
TAC179	Lake Okutama	1989	**55**	31,41,3,3,3,35,37	A	***23***	1,1,19	+	+	+	-	-	-
TAC180	Lake Okutama	1989	**55**	31,41,3,3,3,35,37	A	***23***	1,1,19	+	+	-	-	-	-
TAC187	Lake Okutama	1989	**55**	31,41,3,3,3,35,37	A	***23***	1,1,19	+	+	-	-	-	-
TAC188	Lake Okutama	1989	**55**	31,41,3,3,3,35,37	A	***23***	1,1,19	+	+	-	-	-	-
TAC189	Lake Okutama	1989	**55**	31,41,3,3,3,35,37	A	***23***	1,1,19	+	+	-	-	-	-
TAC190	Lake Okutama	1989	**55**	31,41,3,3,3,35,37	A	***23***	1,1,19	+	+	-	-	-	-
TAC191	Lake Okutama	1989	**55**	31,41,3,3,3,35,37	A	***23***	1,1,19	+	+	-	-	-	-
TAC192	Lake Okutama	1989	**55**	31,41,3,3,3,35,37	A	***23***	1,1,19	+	+	-	-	-	-
TAC122	Lake Barato	1989	**66**	9,47,8,12,9,8,8	A	***28***	4,2,4	+	+	+	-	-	-
TAC123	Lake Barato	1989	**66**	9,47,8,12,9,8,8	A	***28***	4,2,4	+	+	+	-	-	-

Strain	Locality[1]	Year	MLST		Group[2]	*mcy* MLST[3]		Microcystin[4]					
			ST[5]	AP		*m* ST[6]	AP	LR	RR	YR	dLR	dRR	dYR
TAC371	Fukuchi Dam	1990	**72**	36,18,50,39,5,46,43	A	***32***	20,3,15	+	+	-	-	-	-
TAC372	Fukuchi Dam	1990	**72**	36,18,50,39,5,46,43	A	***32***	20,3,15	+	+	-	-	-	-
TAC373	Fukuchi Dam	1990	**72**	36,18,50,39,5,46,43	A	***32***	20,3,15	+	+	-	-	-	-
TAC378	Ishigaki Dam	1990	**72**	36,18,50,39,5,46,43	A	***32***	20,3,15	+	+	-	-	-	-
TAC379	Ishigaki Dam	1990	**72**	36,18,50,39,5,46,43	A	***32***	20,3,15	+	+	+	-	-	-
TAC380	Ishigaki Dam	1990	**72**	36,18,50,39,5,46,43	A	***32***	20,3,15	+	+	+	-	-	-
TAC361	Kunnma Dam	1990	**72**	36,18,50,39,5,46,43	A	***32***	20,3,15	+	+	+	-	-	-
TAC362	Kunnma Dam	1990	**72**	36,18,50,39,5,46,43	A	***32***	20,3,15	+	+	+	-	-	-
TAC363	Kunnma Dam	1990	**72**	36,18,50,39,5,46,43	A	***32***	20,3,15	+	+	-	-	-	-
TAC369	Tatsugami Dam	1990	**72**	36,18,50,39,5,46,43	A	***32***	20,3,15	+	+	+	-	-	-
TAC370	Tatsugami Dam	1990	**72**	36,18,50,39,5,46,43	A	***32***	20,3,15	+	+	-	-	-	-
TAC385	Tengan Dam	1990	**72**	36,18,50,39,5,46,43	A	***32***	20,3,15	+	+	-	-	-	-
TAC386	Tengan Dam	1990	**72**	36,18,50,39,5,46,43	A	***32***	20,3,15	+	+	-	-	-	-
TAC155	Lake Teganuma	1989	**76**	39,54,54,43,49,14,47	A	***34***	21,22,24	+	+	-	-	-	-
TAC157	Lake Teganuma	1989	**76**	39,54,54,43,49,14,47	A	***34***	21,22,24	+	+	-	-	-	-
KA3b	Lake Kasumigaura	2004	**80**	39,56,58,45,51,50,48	A	***36***	22,24,25	+	+	-	-	-	-
Ks05YA11	Lake Kitaura	2005	**92**	39,56,58,50,51,55,48	A	***36***	22,24,25	+	+	-	-	-	-
Tn05AK05	Lake Teganuma	2005	**96**	39,56,68,43,57,55,48	A	***36***	22,24,25	+	+	-	-	-	-
KA4	Lake Kasumigaura	2004	**81**	41,18,59,5,5,16,49	A	***37***	3,3,26	+	+	-	-	-	-
KA6	Lake Kasumigaura	2004	**82**	5,5,5,26,5,11,50	A	***38***	3,25,3	+	-	-	-	-	-
SA2	Lake Suwa	2004	**84**	39,58,61,43,53,51,51	A	***40***	23,26,27	+	-	-	-	-	-
Ks05TA62	Lake Kasumigaura	2005	**87**	39,47,54,43,49,34,48	A	***41***	21,22,25	+	+	-	-	-	-
Ks05IS02	Lake Kasumigaura	2005	**90**	4,61,4,43,4,8,8	A	***43***	1,2,2	+	+	-	-	-	-
Kn05IS01	Lake Kasumigaura	2005	**87**	39,47,54,43,49,34,48	A	***44***	24,22,5	+	+	-	-	-	-
Tn05AK01	Lake Teganuma	2005	**93**	42,16,65,13,56,56,52	A	***45***	25,28,28	+	+	-	-	-	-
In05Fu04	Lake Inba	2005	**97**	44,15,69,50,58,8,54	A	***47***	15,30,29	+	+	-	-	-	-
In05Yo02	Lake Inba	2005	**97**	44,15,69,50,58,8,54	A	***47***	15,30,29	+	+	-	-	-	-
In05Yo05	Lake Inba	2005	**97**	44,15,69,50,58,8,54	A	***47***	15,30,29	+	+	-	-	-	-
In05Yo06	Lake Inba	2005	**97**	44,15,69,50,58,8,54	A	***47***	15,30,29	+	+	-	-	-	-
In05Yo08	Lake Inba	2005	**97**	44,15,69,50,58,8,54	A	***47***	15,30,29	+	+	-	-	-	-
Ia05Yo03	Lake Inba	2005	**98**	30,15,5,50,59,38,55	A	***48***	26,14,5	+	+	-	-	-	-
Ia05Yo05	Lake Inba	2005	**99**	30,15,23,50,59,38,55	A	***49***	26,30,17	+	+	-	-	-	-
Sn05Mb05	Lake Suwa	2005	**100**	36,18,70,26,5,46,56	A	***50***	27,3,15	+	+	-	-	-	-
LNN-s1	Lao P.D.R.	2006	**101**	15,18,18,5,5,11,57	A	***51***	3,31,3	+	-	-	-	-	-
PCC7941	Canada	1954	**21**	14,17,17,14,18,6,15	B	***8***	7,7,8	+	-	-	-	-	-
TAC125	Lake Barato	1989	**31**	20,23,25,19,25,20,6	B	***12***	11,11,11	+	+	-	-	-	-
TAC364	Tatsugami Dam	1990	**33**	6,25,26,20,8,17,6	B	***14***	7,12,12	-	-	-	+	+	+
TAC365	Tatsugami Dam	1990	**33**	6,25,26,20,8,17,6	B	***14***	7,12,12	+	+	-	-	-	-
TAC382	Tengan Dam	1990	**33**	6,25,26,20,8,17,6	B	***14***	7,12,12	-	-	-	+	+	-
TAC67	Rokusuke-ike	1982	**35**	22,27,28,20,8,23,6	B	***15***	12,12,13	+	+	-	-	-	-
TAC69	Rokusuke-ike	1982	**36**	6,28,28,20,6,23,24	B	***16***	12,12,14	+	+	+	-	-	-
NIES298	Lake Kasumigaura	1982	**60**	6,44,42,35,42,23,6	B	***25***	18,18,8	+	-	-	-	-	-
Ks05TA51	Lake Kasumigaura	2005	**86**	35,59,62,47,54,52,25	B	***25***	18,18,8	+	-	-	-	-	-
Ks05IS16	Lake Kasumigaura	2005	**86**	35,59,62,47,54,52,25	B	***25***	18,18,8	+	-	-	-	-	-
Ks05IS11	Lake Kasumigaura	2005	**91**	35,59,62,49,54,54,6	B	***25***	18,18,8	+	-	-	-	-	-
Ks05IS19	Lake Kasumigaura	2005	**102**	35,59,62,47,54,23,25	B	***25***	18,18,8	+	-	-	-	-	-
NIES478	Lake Kasumigaura	1977	**62**	14,17,44,36,43,40,6	B	***26***	7,19,11	+	+	+	-	-	-
TAC95	Lake Barato	1984	**65**	35,23,25,38,44,41,25	B	***27***	19,18,11	+	-	-	-	-	-
TAC355	Kunnma Dam	1990	**68**	6,49,47,20,45,43,6	B	***29***	7,20,21	-	-	-	+	+	+
TAC381	Tengan Dam	1990	**68**	6,49,47,20,45,43,6	B	***29***	7,20,21	-	-	-	+	+	-
TAC356	Kunnma Dam	1990	**69**	20,50,26,20,8,44,6	B	***30***	12,18,22	-	-	-	+	+	-
TAC357	Kunnma Dam	1990	**70**	22,32,48,20,8,45,6	B	***31***	7,20,23	+	+	-	-	-	-
TAC150	Lake Ohnuma	1989	**73**	6,51,51,40,46,47,44	B	***33***	11,21,11	+	+	+	-	-	-
TAC151	Lake Ohnuma	1989	**73**	6,51,51,40,46,47,44	B	***33***	11,21,11	+	+	+	-	-	-

**Strain**	**Locality**[1]	**Year**	**MLST**		**Group**[2]	***mcy* MLST**[3]		**Microcystin**[4]					
			ST[5]	AP		*m* ST[6]	AP	LR	RR	YR	dLR	dRR	dYR
TAC374	Ishigaki Dam	1990	**78**	40,55,56,44,8,39,6	B	***35***	7,23,21	+	+	-	-	-	-
TAC375	Ishigaki Dam	1990	**78**	40,55,56,44,8,39,6	B	***35***	7,23,21	+	+	-	-	-	-
KS1	Lake Kasumigaura	2004	**83**	20,57,60,20,52,45,6	B	***39***	7,23,23	+	+	+	-	-	-
Ki05TA02	Lake Kasumigaura	2005	**88**	14,27,63,48,55,53,25	B	***42***	11,27,11	-	-	-	+	-	-
Ki05TA07	Lake Kasumigaura	2005	**89**	6,60,64,48,55,53,25	B	***42***	11,27,11	+	-	-	-	-	-
TAC159	Lake Teganuma	1989	**48**	20,37,36,8,36,33,24	B	***18****	14,13,16	-	-	-	-	-	-
TAC160	Lake Teganuma	1989	**48**	20,37,36,8,36,33,24	B	***18****	14,13,16	-	-	-	-	-	-
TAC185	Lake Teganuma	1989	**48**	20,37,36,8,36,33,24	B	***18****	14,13,16	-	-	-	-	-	-
TAC134	Lake Suwa	1989	**51**	20,38,38,20,36,20,35	B	***20****	16,15,8	-	-	-	-	-	-
NIES98	Lake Kasumigaura	1982	**6**	6,6,6,6,6,6,6	B	-	-	-	-	-	-	-	-
NIES99	Lake Suwa	1982	**7**	6,7,6,6,6,6,6	B	-	-	-	-	-	-	-	-
NIES101	Lake Suwa	1982	**9**	8,7,6,6,8,6,6	B	-	-	-	-	-	-	-	-
TAC50	Lake Suwa	1982	**9**	8,7,6,6,8,6,6	B	-	-	-	-	-	-	-	-
TAC51	Lake Suwa	1982	**9**	8,7,6,6,8,6,6	B	-	-	-	-	-	-	-	-
TAC76	Lake Yogo	1984	**37**	23,29,29,22,27,24,25	B	-	-	-	-	-	-	-	-
TAC115	Nepal	1988	**40**	24,30,31,23,30,25,27	B?	-	-	-	-	-	-	-	-
TAC128	Kamisawa-ike	1989	**42**	26,32,33,25,32,27,29	B	-	-	-	-	-	-	-	-
TAC136	Showa-tameike	1989	**44**	27,33,26,27,33,29,6	B	-	-	-	-	-	-	-	-
TAC146	Lake Ohnuma	1989	**45**	26,34,33,28,32,30,31	B	-	-	-	-	-	-	-	-
NIES299	Lake Kasumigaura	1979	**61**	20,45,43,36,33,39,6	B	-	-	-	-	-	-	-	-
TAC74	Lake Yogo	1984	**63**	20,46,45,37,33,24,6	B	-	-	-	-	-	-	-	-
NIES91	Lake Kasumigaura	1982	**77**	20.7,55,6,50,6,6	B	-	-	-	-	-	-	-	-
T20-3	Thailand	1996	**23**	16,19,19,15,20,15,17	X	***10***†	9,9,9	-	-	-	+	+	-
TAC170	Lake Okutama	1989	**57**	33,43,40,34,41,37,39	X	***24***†	17,17,20	+	+	-	-	-	-
Tn05AK03	Lake Teganuma	2005	**95**	33,43,67,20,41,57,53	X	***46***†	17,29,20	+	-	-	-	-	-
NIES109	Lake Yogo	1982	**15**	12,13,12,11,13,13,11	C	-	-	-	-	-	-	-	-
NIES604	Lake Kasumigaura	1977	**17**	12,14,14,11,15,13,12	C	-	-	-	-	-	-	-	-
TAC395	Lake Suwa	1990	**17**	12,14,14,11,15,13,12	C	-	-	-	-	-	-	-	-
TAC198	Lake Barato	1990	**50**	12,14,37,32,38,13,34	C	-	-	-	-	-	-	-	-
TAC199	Lake Barato	1990	**50**	12,14,37,32,38,13,34	C	-	-	-	-	-	-	-	-
TAC200	Lake Barato	1990	**50**	12,14,37,32,38,13,34	C	-	-	-	-	-	-	-	-
TAC124	Lake Barato	1989	**67**	12,48,46,11,15,42,41	C	-	-	-	-	-	-	-	-
NIES100	Lake Suwa	1982	**8**	7,8,7,7,7,7,7	D	-	-	-	-	-	-	-	-
TAC401	Lake Suwa	1990	**8**	7,8,7,7,7,7,7	D	-	-	-	-	-	-	-	-
TAC402	Lake Suwa	1990	**8**	7,8,7,7,7,7,7	D	-	-	-	-	-	-	-	-
TAC15	Lake Kasumigaura	1978	**24**	7,8,20,7,21,16,18	D	-	-	-	-	-	-	-	-
TAC20	Lake Kasumigaura	1978	**24**	7,8,20,7,21,16,18	D	-	-	-	-	-	-	-	-
TAC19	Lake Kasumigaura	1978	**25**	7,8,20,7,21,17,18	D	-	-	-	-	-	-	-	-
TAC65	Chitsato-ike	1982	**27**	7,8,22,7,22,16,19	D	-	-	-	-	-	-	-	-
TAC147	Lake Ohnuma	1989	**27**	7,8,22,7,22,16,19	D	-	-	-	-	-	-	-	-
TAC148	Lake Ohnuma	1989	**27**	7,8,22,7,22,16,19	D	-	-	-	-	-	-	-	-
TAC149	Lake Ohnuma	1989	**27**	7,8,22,7,22,16,19	D	-	-	-	-	-	-	-	-
TAC66	Rokusuke-ike	1982	**27**	7,8,22,7,22,16,19	D	-	-	-	-	-	-	-	-
TAC98	Lake Shirakaba	1984	**39**	7,8,30,7,29,17,26	D	-	-	-	-	-	-	-	-
TAC173	Lake Shirakaba	1989	**39**	7,8,30,7,29,17,26	D	-	-	-	-	-	-	-	-
TAC174	Lake Shirakaba	1989	**39**	7,8,30,7,29,17,26	D	-	-	-	-	-	-	-	-
TAC96	Lake Shirakaba	1984	**39**	7,8,30,7,29,17,26	D	-	-	-	-	-	-	-	-
TAC396	Lake Suwa	1990	**53**	7,39,7,7,7,7,7	D	-	-	-	-	-	-	-	-
NIES901	Britain	1997	**59**	7,8,41,7,29,38,18	D	-	-	-	-	-	-	-	-
TAC75	Lake Yogo	1984	**64**	7,8,22,7,22,17,40	D	-	-	-	-	-	-	-	-
TAC376	Ishigaki Dam	1990	**71**	7,8,49,7,29,17,42	D	-	-	-	-	-	-	-	-
TAC377	Ishigaki Dam	1990	**71**	7,8,49,7,29,17,42	D	-	-	-	-	-	-	-	-
TAC358	Kunnma Dam	1990	**71**	7,8,49,7,29,17,42	D	-	-	-	-	-	-	-	-

Strain	Locality[1]	Year	MLST		Group[2]	*mcy* MLST[3]		Microcystin[4]					
			ST[5]	AP		*m* ST[6]	AP	LR	RR	YR	dLR	dRR	dYR
TAC359	Kunnma Dam	1990	**71**	7,8,49,7,29,17,42	D	-	-	-	-	-	-	-	-
TAC360	Kunnma Dam	1990	**71**	7,8,49,7,29,17,42	D	-	-	-	-	-	-	-	-
TAC368	Tatsugami Dam	1990	**71**	7,8,49,7,29,17,42	D	-	-	-	-	-	-	-	-
TAC387	Maesato Dam	1990	**79**	7,8,57,7,22,17,26	D	-	-	-	-	-	-	-	-
TAC388	Maesato Dam	1990	**79**	7,8,57,7,22,17,26	D	-	-	-	-	-	-	-	-
TAC389	Maesato Dam	1990	**79**	7,8,57,7,22,17,26	D	-	-	-	-	-	-	-	-
TAC383	Tengan Dam	1990	**79**	7,8,57,7,22,17,26	D	-	-	-	-	-	-	-	-
TAC384	Tengan Dam	1990	**79**	7,8,57,7,22,17,26	D	-	-	-	-	-	-	-	-
NIES44	Lake Kasumigaura	1974	**1**	1,1,1,1,1,1,1	E	-	-	-	-	-	-	-	-
NIES110	Lake Kasumigaura	1978	**1**	1,1,1,1,1,1,1	E	-	-	-	-	-	-	-	-
TAC39	Lake Kasumigaura	1978	**1**	1,1,1,1,1,1,1	E	-	-	-	-	-	-	-	-
TAC40	Lake Kasumigaura	1978	**1**	1,1,1,1,1,1,1	E	-	-	-	-	-	-	-	-
NIES106	Lake Kasumigaura	1982	**1**	1,1,1,1,1,1,1	E	-	-	-	-	-	-	-	-
TAC163	Lake Teganuma	1989	**1**	1,1,1,1,1,1,1	E	-	-	-	-	-	-	-	-
TAC164	Lake Teganuma	1989	**1**	1,1,1,1,1,1,1	E	-	-	-	-	-	-	-	-
NIES105	Lake Kasumigaura	1982	**12**	11,11,10,1,11,10,1	E	-	-	-	-	-	-	-	-
NIES111	Lake Kasumigaura	1978	**14**	11,12,11,1,12,12,1	E	-	-	-	-	-	-	-	-
NIES108	Lake Suwa	1982	**14**	11,12,11,1,12,12,1	E	-	-	-	-	-	-	-	-
TAC57	Lake Suwa	1982	**14**	11,12,11,1,12,12,1	E	-	-	-	-	-	-	-	-
NIES112	Lake Suwa	1982	**16**	11,12,13,1,14,1,1	E	-	-	-	-	-	-	-	-
NIES904	Thailand	1996	**54**	11,40,13,1,39,1,1	E	-	-	-	-	-	-	-	-
TAC171	Lake Okutama	1989	**58**	34,1,11,1,39,1,1	E	-	-	-	-	-	-	-	-
TAC172	Lake Okutama	1989	**58**	34,1,11,1,39,1,1	E	-	-	-	-	-	-	-	-
TAC91	Lake Barato	1984	**2**	2,2,2,2,2,2,2	NA	-	-	-	-	-	-	-	-
NIES87	Lake Kasumigaura	1982	**2**	2,2,2,2,2,2,2	NA	-	-	-	-	-	-	-	-
NIES104	Chiyodaku	1982	**11**	10,10,9,9,10,9,9	NA	-	-	-	-	-	-	-	-
TAC114	Nepal	1988	**30**	19,22,24,18,24,19,22	NA	-	-	-	-	-	-	-	-
TAC4	Lake Kasumigaura	1978	**34**	21,26,27,21,26,22,23	NA	-	-	-	-	-	-	-	-
TAC6	Lake Kasumigaura	1978	**34**	21,26,27,21,26,22,23	NA	-	-	-	-	-	-	-	-
TAC110	Shigure Dam	1984	**38**	2,2,2,2,28,2,2	NA	-	-	-	-	-	-	-	-
TAC126	Shin-ike	1989	**41**	25,31,32,24,31,26,28	NA	-	-	-	-	-	-	-	-
TAC152	Lake Ohnuma	1989	**46**	28,35,25,29,34,31,31	NA	-	-	-	-	-	-	-	-
TAC153	Lake Ohnuma	1989	**46**	28,35,25,29,34,31,31	NA	-	-	-	-	-	-	-	-
TAC352	Lake Touhutsu	1990	**47**	29,36,35,30,35,32,33	NA	-	-	-	-	-	-	-	-
TAC169	Lake Okutama	1989	**56**	32,42,39,33,40,36,38	NA	-	-	-	-	-	-	-	-
TAC156	Lake Teganuma	1989	**74**	37,52,52,41,47,48,45	NA	-	-	-	-	-	-	-	-
TAC154	Lake Teganuma	1989	**75**	38,53,53,42,48,49,46	NA	-	-	-	-	-	-	-	-

Footnotes

[1] Geographic location of NIES and TAC strains are available at http://mcc.nies.go.jp/distribution-phylogeny_e.html.

[2] Group assignment based on the MLST phylogeny in [13] and Fig. 3a. NA, strains assigned to neither group.

[3] Microcystin variants are abbreviated as follows: LR, microcystin-LR; RR, microcystin-RR; YR, microcystin-YR; dLR, [Dha[7]]microcystin-LR; dRR, [Dha[7]]microcystin-RR; dYR, [Dha[7]]microcystin-YR.

[4] Sequence type (ST). Note that the group assignment of ST40 is ambiguous.

[5] Allelic profile of MLST presented in the following order: *ftsZ* , *glnA* , *gltX* , *gyrB* , *pgi* , *recA* , *tpi* .

[6] *mcy* sequence types (*mcy* ST) are indicated in italic to avoid the confusion with the STs of MLST. * Non-microcystin producing *mcy* STs. † *mcy* STs that showed discordant phylogenetic relationships between different *mcy* loci.

[7] Allelic profile of *mcy* MLST presented in the following order: *mcyD* , *mcyG* , *mcyJ* .

[8] The type strain of *M. aeruginosa* .
